# Lever sign test for anterior cruciate ligament injuries: a diagnostic meta-analysis

**DOI:** 10.1186/s13018-024-04635-w

**Published:** 2024-03-01

**Authors:** Shiqiang Hu, Xiaoping Wang, Qiyue Wang, Weili Feng

**Affiliations:** 1 Orthopaedics Department, Xiaolan People’s Hospital of Zhongshan, Zhongshan, People’s Republic of China; 2 Sports Medicine Center, Department of Orthopaedics Surgery, First Affiliated Hospital of Shantou University Medical College, Shantou, People’s Republic of China

**Keywords:** Anterior cruciate ligament, Lever sign test, Diagnostic, Meta-analysis

## Abstract

**Background:**

Sports-related ACL (anterior cruciate ligament) injuries are frequent. Successful management requires early diagnosis and treatment. One of the clinical tests used to identify ACL damage is the lever sign test. This meta-analysis aimed to assess the lever sign test's diagnostic efficacy for ACL injuries.

**Methods:**

An extensive investigation of the Cochrane Library, Embase, and PubMed databases was conducted until April 2023. Studies assessing the lever sign test's diagnostic efficacy for ACL injuries were also included. A bivariate random-effects model was employed to acquire the pooled estimates of diagnostic odds ratios, specificity, positive and negative likelihood ratios, sensitivity, and curves of the summary receiver operating characteristic (SROC).

**Results:**

The meta-analysis comprised twelve investigations with a total of 1365 individuals. The lever sign test's combined sensitivity and specificity for the purpose of diagnosing injuries to the ACL were 0.810 (95% confidence interval [CI] 0.686–0.893) and 0.784 (95% CI 0.583–0.904), respectively. The positive and negative likelihood ratios were 3.148 (95% CI 1.784–5.553) and 0.210 (95% CI 0.084–0.528), respectively. The study revealed a diagnostic odds ratio of 17.656, with a 95% CI ranging from 4.800 to 64.951. The SROC curve's area was determined to be 0.912 (95% CI 0.857–0.967).

**Conclusion:**

With high specificity and sensitivity, the lever sign test is a reliable diagnostic modality for ACL injuries. However, the test should be used in combination with other diagnostic tests to increase the accuracy of the diagnosis. Further investigations are warranted to assess the clinical practicability of the lever sign test in various populations and settings.

## Introduction

Anterior cruciate ligament (ACL) injury is a prevalent and significant sports-related injury, with an incidence of about 250,000 cases every year in the US as a whole [1]. ACL injuries can lead to significant morbidity and decreased quality of life, with long-term consequences such as osteoarthritis and reduced physical activity levels [2]. Early diagnosis and management of ACL injuries are crucial for successful treatment and rehabilitation.

**Fig. 1:**
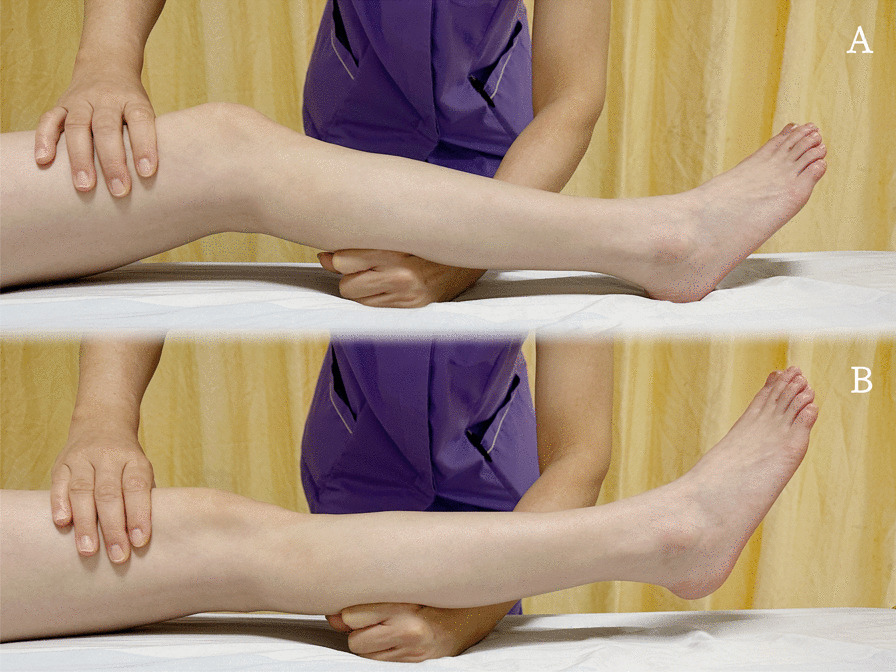
This figure expresses two diagrams that demonstrate the lever sign test, which is used to diagnose ACL ruptures. The test involves placing a fist under the patient's calf and applying pressure to their quadriceps with a second hand. **A** shows a positive Lever Sign test. The physician applies the pressure to the quadriceps with one hand, while the other hand serves as the fulcrum. In this case, the ACL has been ruptured and is unable to counteract the force of gravity. As a result, the foot remains on the examination table despite the pressure being applied. **B** shows a negative lever sign test. Like in **A**, pressure is applied to the quadriceps with the second hand. However, in this case, the ACL is intact and is able to counteract the force of gravity. Therefore, the ACL is able to counteract the downward force and keep the foot raised

The lever sign test is increasingly utilized as a prevalent clinical assessment tool for the purpose of diagnosing injuries of ACL [3]. The procedure is to place the patient in a position of supine lying, in which the individual lies on their back while extending their lower limbs. The examiner stands beside the subject and places a closed fist under the proximal third of the calf while simultaneously applying downward pressure to the anterior thigh. In cases where the ACL remains intact, the tibia will move forward in relation to the femur when pressure is applied, accompanied by the heel leaving the bed, and the patient is expected to be free from pain or discomfort. However, if the ACL is partially or completely torn, the lever created by the ligament resisting gravity will be compromised. This leads to anterior translation of the tibial plateau relative to the femoral condyle, preventing the heel from leaving the bed, and may result in pain or discomfort for the patient. Figure 1 illustrates the manipulation of the lever sign test.

Despite the widespread lever sign test clinical application, its diagnostic efficacy lacks systematical evaluation. Several studies have reported on the lever sign test specificity and sensitivity for diagnosing ACL injuries, but the results have been inconsistent [4–6]. A meta-analysis can provide a more accurate detection of the diagnostic efficacy of the lever sign test by pooling the multiple studies' outcomes [7]. The objective of the investigation was to conduct a meta-analysis in order to detect the accuracy of the lever sign test when diagnosing injuries of ACL.

## Methods

The present meta-analysis was performed following the PRISMA 2020 rules, which are the preferred reporting items for systematic reviews and meta-analyses [8]. The investigation has been properly recorded with PROSPERO [9], with the identification number CRD42022339218.

### Literature search

A comprehensive review of the literature was performed utilizing the Embase, PubMed, and Cochrane Library databases from their inception until March 2023. The following different combinations of search terms were utilized: "anterior cruciate ligament," "ACL," "lever sign test," "diagnostic," "sensitivity," "specificity," and "accuracy." Only articles published in English and involving human subjects were included. Additional relevant studies have been determined by screening the reference lists of the detected articles.

### Study selection

The inclusion criteria for the selected studies were as follows: (1) The study aimed to evaluate the Lever Sign test efficacy in diagnosing injuries of the ACL; (2) the investigation provided adequate data to construct 2 × 2 contingency tables of true positives (TP), false positives (FP), true negatives (TN), and false negatives (FN); (3) the study used arthroscopic or MRI examination as the reference standard; (4) the study was conducted in humans; (5) the investigation was published in English.

Investigations were excluded if they dropped in the following specifications: (1) the study was a review article, conference abstract, or case report; (2) the investigation did not report primary data; (3) the study did not report the lever sign test accuracy in diagnosis; (4) the study did not use arthroscopic or MRI examination as the reference standard; (5) the study was conducted in animals or cadavers.

The eligibility of the studies selected was screened by both authors (SH, XW) through an independent assessment of the titles and abstracts. The same two authors retrieved and reviewed full-text articles of potentially relevant studies independently. Discussions with the corresponding author helped to overcome conflicts.

### Extraction of data and quality assessment

The data extraction process was performed by two authors (XW, SH) in a manner that ensured independence, utilizing a standardized form for the purpose. The following information was extracted: publication years, study population features, design of the study, criteria of exclusion and inclusion, diagnostic criteria for ACL injury, lever sign test methodology, reference standard, and diagnostic performance data.

The authors QW and XW conducted an independent evaluation of the studies' quality employing the revised Quality Assessment of Diagnostic Accuracy Studies (QUADAS-2) tool [10]. The tool is comprised of four distinct domains, namely patient selection, index test, reference standard, and flow and timing. The bias risk for each domain is evaluated, and the first three domains are additionally evaluated for concerns related to applicability. Any disagreements in the quality evaluation were fixed by discussing the corresponding author.

### Data synthesis and analysis

To evaluate the efficacy of the lever sign test in ACL injury detection, MRI or arthroscopy was used as a reference standard. Data on the TP, FP, FN, and TN were obtained independently by two reviewers (XW, QW) from the involved manuscripts. If only specificity and sensitivity were obtainable, the calculation of these numbers was conducted utilizing Web-based tools. Articles that did not provide such data were excluded from the study.

The presence of heterogeneity in meta-analyses of diagnostic accuracy is a frequent occurrence; therefore, the utilization of random effects models is standard. The aforementioned models possess the capability to approximate the mean accuracy of the examination and clarify the variations in this outcome. Due to its inability to account for threshold effects, the traditional I^2^ statistics is not adopted for quantifying heterogeneity in sensitivity and specificity. Exploratory analyses were conducted by visually examining forest and SROC plots to assess whether factors were associated with test accuracy.

A bivariate random-effects model was utilized to conduct a meta-analysis aimed at estimating the combined sensitivity and specificity of the lever sign test in the diagnosis of ACL tears. The utilization of the bivariate model enables the incorporation of the correlation existing across sensitivity and specificity, thereby facilitating the computation of summary receiver operating characteristic (SROC) curves. With values ranging from 0.5 (no discrimination) to 1.0 (perfect discrimination), the area under the curve (AUC) was utilized as a marker of overall diagnostic efficacy.

Analyses were conducted utilizing Onlinemeta v1.0: 2022.3.15 (https://smuonco.Shinyapps.io/Onlinemeta/) [11] and Meta-DiSc (www.metadisc.es.) [12].

## Results

### Quality of included studies and methodological heterogeneity

Table 1 indicates that the studies conducted had average to high quality. All studies involved patients who were typically diagnosed with anterior cruciate ligament injury through MRI or arthroscopy. Deveci et al. [13] fail to present the original data, making it unable to calculate the TP, FP, FN, and TN. Therefore, this study was not included in the quantitative analysis though demonstrated in the table. Each study was a cohort study, which reduced the possibility of patient selection bias. However, seven of the investigations did not clearly specify whether blinding was implemented in the patient selection process and the intervention process [4, 5, 14–18]. The examiners who performed the lever sign test varied in countries and regions. Two studies subdivided the lever sign test performed pre-anesthesia and post-anesthesia, as described by Deveci et al. and Chong et al. [13, 14]. Four investigations included patients with acute ACL injuries (less than 1 month) [4, 5, 17, 19] while another two studies recruited individuals experiencing chronic ACL tears (more than 1 month) [13, 20]. Lelli et al. enlisted both acute and chronic ACL injury participants [3]. Six articles did not specify whether the subjects had an acute or chronic ACL injury [14–16, 18, 21, 22]. The studies incorporated subjects primarily comprising young adults and adolescents, in accordance with the epidemiology of ACL injuries [23].

**Table 1:** Characteristics of included studies

No.	Study	Year	Country	Study design	Blindness	Participant	No. of participants	Age (years)
1	Lelli	2014	Italy	Cohort	Yes	Patients with a definitive MRI diagnosis of unilateral ACL rupture (partial or complete)	281 males 119 females	26.43 ± 14.9
2	Deveci	2015	Turkey	Cohort	Yes	Patients diagnosed with ACL tear which was definitively determined during an arthroscopic surgical procedure	96 males 21 females	25.8 ± 5.9 (17–45)
3	Chong	2017	USA	Cohort	NM	Patients who presented to the lead orthopedic surgeon with a unilateral knee injury that resulted in symptomatic instability at two selected facilities	21 males 12 females	Male:30.9 ± 14.3 (11–62) female: 30.6 ± 17.0 (15–60)
4	Mulligan	2017	USA	Cohort	Yes	Patients with a complaint of knee pain rated as less than 7/10 on a verbal numerical rating scale, possessing at least 20–120° range of motion	38 males 22 females	42 ± 13.4 (18–65)
5	Thapa	2015	Nepal	Cohort	NM	Patients with knee symptoms of giving way/locking/pain following sports or non sports injury	50 males 30 females	Mean 32.12 (21–42)
6	Massey	2017	USA	Cohort	Yes	Presenting after a noncontact or contact knee injury with subjective swelling, or an objective effusion, and uninjured normal contralateral knee for comparison (no previous injury or surgery)	61 males 30 females	28 ± 7
7	Jarbo	2017	USA	Cohort	Yes	Patients with a chief concern of acute knee pain who came for an evaluation within 4 weeks of their injury or the onset of symptoms	58 males 44 females	Mean 23 (15–66)
8	Lichtenberg	2018	Netherland	Cohort	NM	Patients ≥ 16 years old, suffered from knee trauma, and had indications for knee arthroscopic surgery	57 males 37 females	34 ± 15
9	Polat	2019	Turkey	Cohort	NM	Patients who had contact or noncontact knee injuries up to 2 weeks prior to the examination and who did not have any previous history of knee injury	69 males 9 females	26.2 ± 6.4 (17–44)
10	McQuivey	2019	USA	Cohort	NM	Patients with probable acute ACL tears without other previous or simultaneous knee pathology in patients ages 12–55 years	25 males 20 females	Mean 33 (12–54)
11	Marcel	2020	Brazil	Cohort	Yes	Patients with a history of previous knee sprains	49 males 23 femals	33.2 ± 8.6
12	Kevin	2021	Belgium	Cohort	NM	Patients above 18 years, presenting at the ED for acute knee pain, following an acute trauma within 8 days, with an initial radiograph showing no signs of fracture (except Segond fracture or tibial spine fracture)	52 (with a 2 M/1F sex distribution)	Mean 33 (19–56)
13	Camille	2021	France	Cohort	NM	Patients aged 18 and over who were living in France, recent unilateral knee injury (less than 7 days) with clinical suspicion of ACL injury (cracking, instability, apprehension, effusion) and a normal X-ray	68 males 190 femals	42.2 ± 13.4

No.	Study	Trauma period	Gold standard	Sample size	Sensitivity (%)	Specificity (%)	TP	FP	FN	TN
1	Lelli	Acute: ≤ 20 days chronic: > 20 days	MRI	Lever Sign test: 100	100.00	100.00	400	0	0	0
				Lachman test: 100	62.00	100.00	247	0	153	0
				Anterior Drawer test: 100	72.00	100.00	287	0	113	0
				Pivot Shift test: 100	47.00	100.00	188	0	212	0
2	Deveci	8.7 weeks (4–25 weeks)	MRI & arthroscopy	Lever Sign test (pre-anaesthesia): 117	94.00	UTC	UTC	UTC	UTC	UTC
				Lachman test (pre-anaesthesia): 117	80.00	UTC	UTC	UTC	UTC	UTC
				Anterior Drawer test (pre-anaesthesia): 117	60.00	UTC	UTC	UTC	UTC	UTC
				Pivot Shift test (pre-anaesthesia): 117	62.00	UTC	UTC	UTC	UTC	UTC
				Lever Sign test (under anaesthesia): 117	98.00	UTC	UTC	UTC	UTC	UTC
				Lachman test (under anaesthesia): 117	88.00	UTC	UTC	UTC	UTC	UTC
				Anterior Drawer test (under anaesthesia): 117	88.00	UTC	UTC	UTC	UTC	UTC
				Pivot Shift test (under anaesthesia): 117	88.00	UTC	UTC	UTC	UTC	UTC
3	Chong	NM	Arthroscopy	Lever Sign test (pre-anaesthesia, EOS): 33	88.00	UTC	29	0	4	0
				Lachman test (pre-anaesthesia, EOS): 33	94.00	UTC	31	0	2	0
				Pivot Shift test (pre-anaesthesia, EOS): 33	27.00	UTC	11	0	24	0
				Lever Sign test (under anaesthesia, EOS): 33	97.00	UTC	32	0	1	0
				Lachman test (under anaesthesia, EOS): 33	100.00	UTC	33	0	0	0
				Pivot Shift test (under anaesthesia, EOS): 33	97.00	UTC	32	0	1	0
				Lever Sign test (pre-anaesthesia, EOPA): 33	82.00	UTC	27	0	6	0
				Lachman test (pre-anaesthesia, EOPA): 33	67.00	UTC	22	0	11	0
				Pivot Shift test (pre-anaesthesia, EOPA): 33	9.00	UTC	3	0	30	0
				Lever Sign test (under anaesthesia, EOPA): 33	100.00	UTC	33	0	0	0
				Lachman test (under anaesthesia, EOPA): 33	94.00	UTC	31	0	2	0
				Pivot Shift test (under anaesthesia, EOPA): 33	76.00	UTC	25	0	8	0
4	Mulligan	NM	Injury history interview or review of previously conducted radiographic or MRI	Lever Sign test (direct visual assessment of ACL): 19	33.00	50.00	5	2	10	2
				Lever Sign test (application of clinical cluster of findings): 41	44.00	75.00	4	8	5	24
5	Thapa	NM	Arthroscopy	Lever Sign test: 80	85.71	91.11	30	5	5	40
				Lachman test: 80	91.42	95.55	32	2	3	43
				Pivot shift test: 80	51.42	100.00	18	0	17	45
				Anterior drawer test: 80	80.00	93.33	28	3	7	42
6	Massey	NM	MRI	Lever Sign test: 91	83.00	80.00	59	4	12	16
				Lachman test: 91	89.00	85.00	63	3	8	17
				Pivot shift test: 83	66.00	94.00	44	1	23	15
				Anterior drawer test: 91	82.00	80.00	58	4	13	16
7	Jarbo	≤ 4 weeks	MRI & arthroscopy	Lever Sign test: 102	63.00	90.00	32	5	19	46
				Lachman test: 102	90.00	96.00	46	2	5	49
				Pivot shift test: 102	59.00	98.00	29	1	20	52
				Anterior drawer test: 102	88.00	94.00	45	3	6	48
8	Lichtenberg	NM	Arthroscopy	Lever Sign test: 87	39.00	100.00	16	0	25	46
				Lachman test: 93	87.00	91.00	40	4	6	43
				Pivot shift test: 81	50.00	98.00	20	1	20	40
				Anterior drawer test: 91	71.00	94.00	39	2	16	34
9	Polat	≤ 2 weeks	MRI	Lever Sign test (acute): 78	91.90	93.80	57	1	5	15
				Lachman test (acute): 78	80.60	62.50	50	6	12	10
				Pivot shift test (acute): 78	51.60	93.80	32	1	30	15
				Anterior drawer test (acute): 78	77.40	68.80	48	5	14	11
				Lever Sign test (preanesthesia): 78	91.90	93.80	57	1	5	15
				Lachman test (preanesthesia): 78	83.90	68.80	52	5	13	12
				Pivot shift test (preanesthesia): 78	56.50	93.80	35	1	27	15
				Anterior drawer test (preanesthesia): 78	79.00	75.00	49	4	13	12
10	McQuivey	NM	MRI	Lever Sign test: 21	100.00	93.80	5	1	0	15
				Lachman test /Anterior drawer test: 24	40.00	100.00	6	0	9	9
11	Marcel	> 1 months	MRI	Lachman test (without anesthesia): 72	94.80	100.00	55	0	3	14
				Anterior Drawer test (without anesthesia): 72	82.00	84.85	32	5	7	28
				Lever Sign test (without anesthesia): 72	64.10	100.00	41	0	8	23
12	Kevin	≤ 8 days	MRI	Lever Sign test: 52	92.50	25.00	37	9	3	3
				Lachman test: 52	54.00	54.50	22	5	19	6
				Anterior Drawer test: 52	56.00	82.00	23	2	18	9
13	Camille	≤ 7 days	MRI	Lever Sign test: 258	61.20	27.80	134	26	85	10
				Lachman test: 258	99.10	5.60	217	34	2	2

TP, True Positive; FP, False Positive; FN, False Negative; TN, True Negative; UTC, unable to calculate; NM, not mentioned; EOS, experienced orthopedic surgeon; EOPA, experienced orthopedic physician assistant; ACL, anterior cruciate ligament

### Study characteristics

**Fig. 2:**
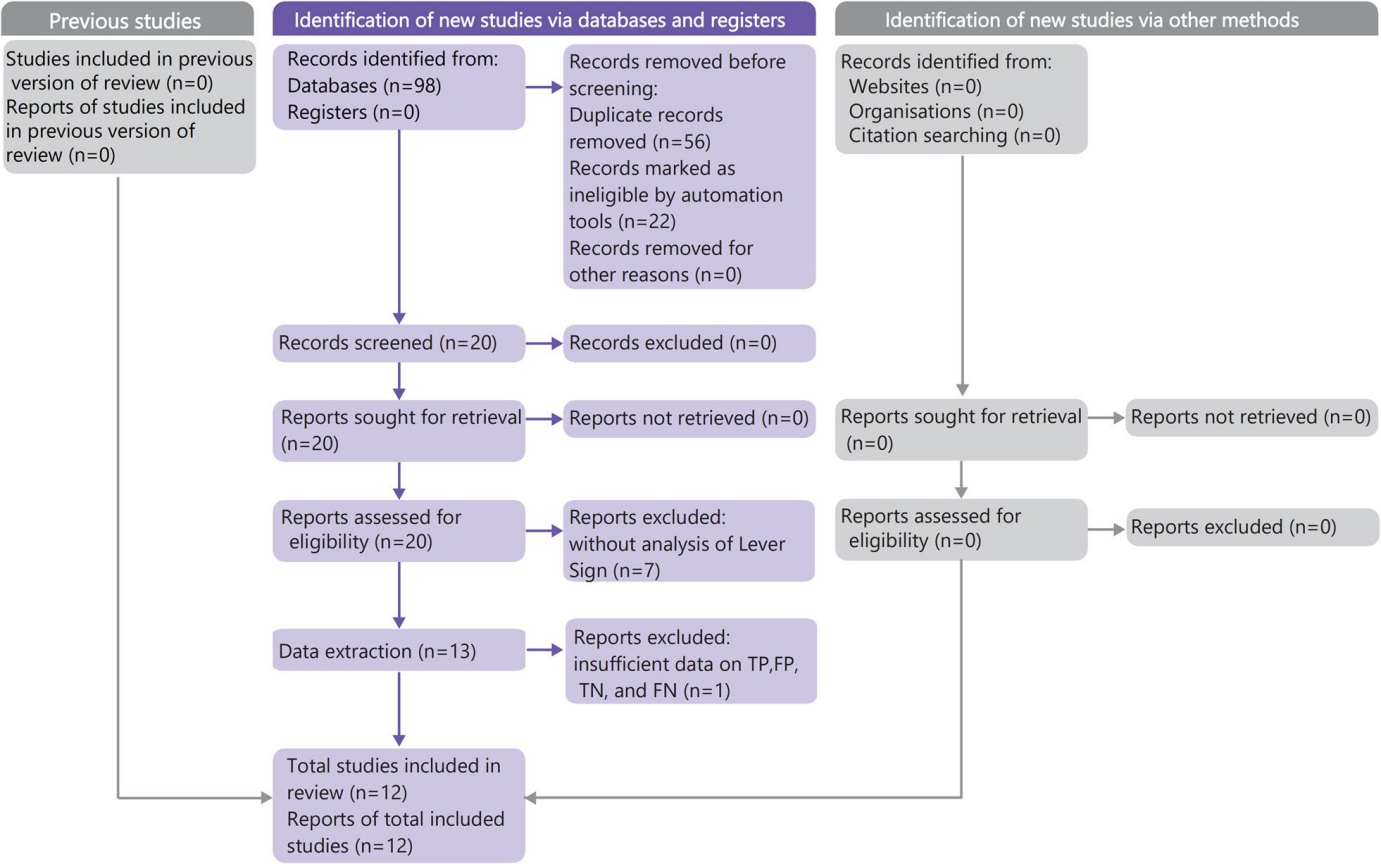
Flowchart of articles retrieved from search of databases and other resource with reasons of exclusion

Ninety-eight articles were initially identified through a literature search, out of which twenty were deemed possibly eligible according to the screening of their titles and abstracts. Following a comprehensive examination of the texts, a total of 13 articles were found to satisfy the established criteria for inclusion. The meta-analysis consisted of 12 studies, with one article being excluded from the meta-analysis owing to insufficient data on TP, FP, TN, and FN. The overall number of participants included in the meta-analysis was 1365, with 811 being male and 554 being female. Figure 2 illustrates the flowchart detailing the procedure of study selection. Table 2 demonstrates the features of the investigations that have been incorporated.

**Table 2:** QUADAS-2 results

Study	Risk of bias				Applicability concerns		
	Patient selection	Index test	Reference standard	Flow and timing	Patient selection	Index test	Reference standard
Study 1	☺	?	☺	☺	☺	☺	☺
Study 2	?	☺	?	?	☺	☺	☺
Study 3	☹	☺	☺	☺	☺	☺	☺
Study 4	☺	☹	?	?	☺	☺	☺
Study 5	?	☹	☺	☺	?	☺	☺
Study 6	☺	☺	☺	☺	☺	☺	?
Study 7	?	☺	?	?	☺	?	☺
Study 8	☺	☺	?	?	?	☺	☺
Study 9	☺	☹	?	?	?	☺	☺
Study 10	☺	☹	?	?	☺	☺	☺
Study 11	☺	☹	?	?	☺	☺	☺
Study 12	☺	☹	?	?	☺	☺	☺

☺ low risk, ☹ high risk, ? unclear risk

### Meta-analysis

**Fig. 3:**
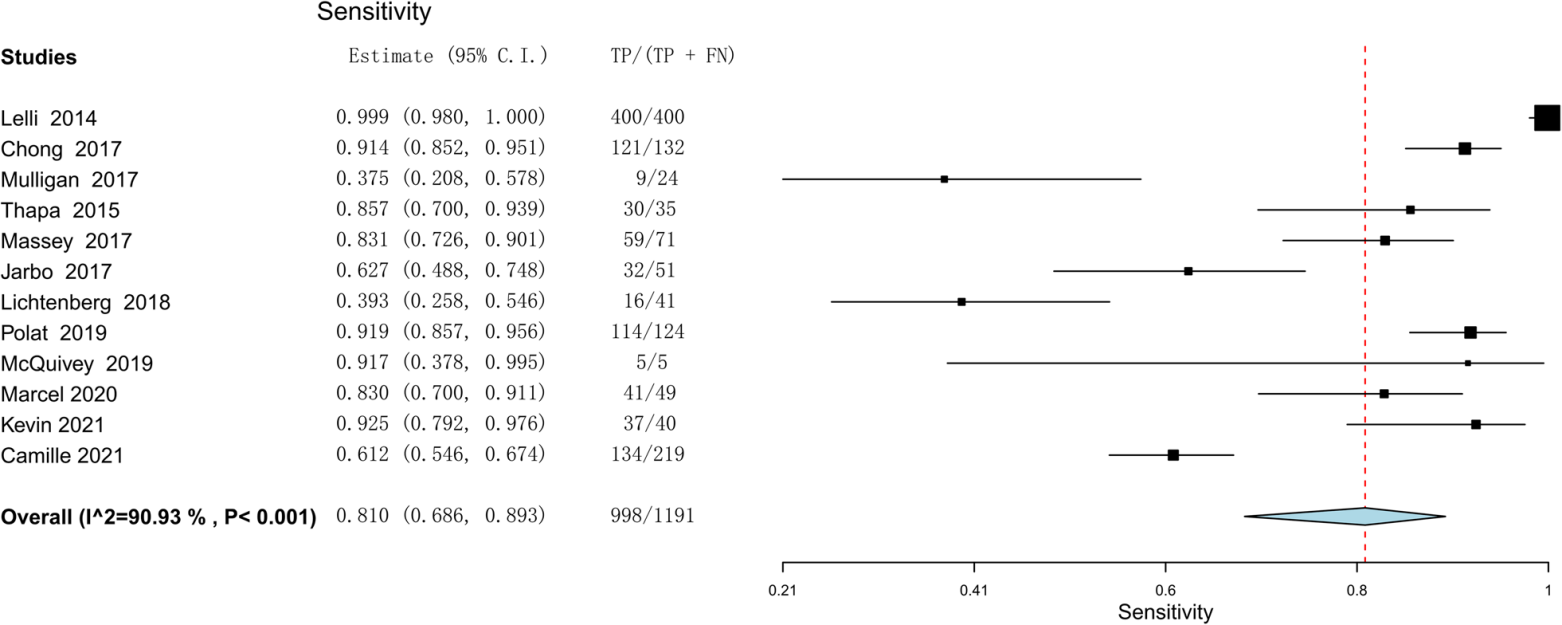
Sensitivity part of the coupled forrest plot for the lever sign test compared to MRI/arthroscopy, for the diagnosis of anterior cruciate ligament injuries

**Fig. 4:**
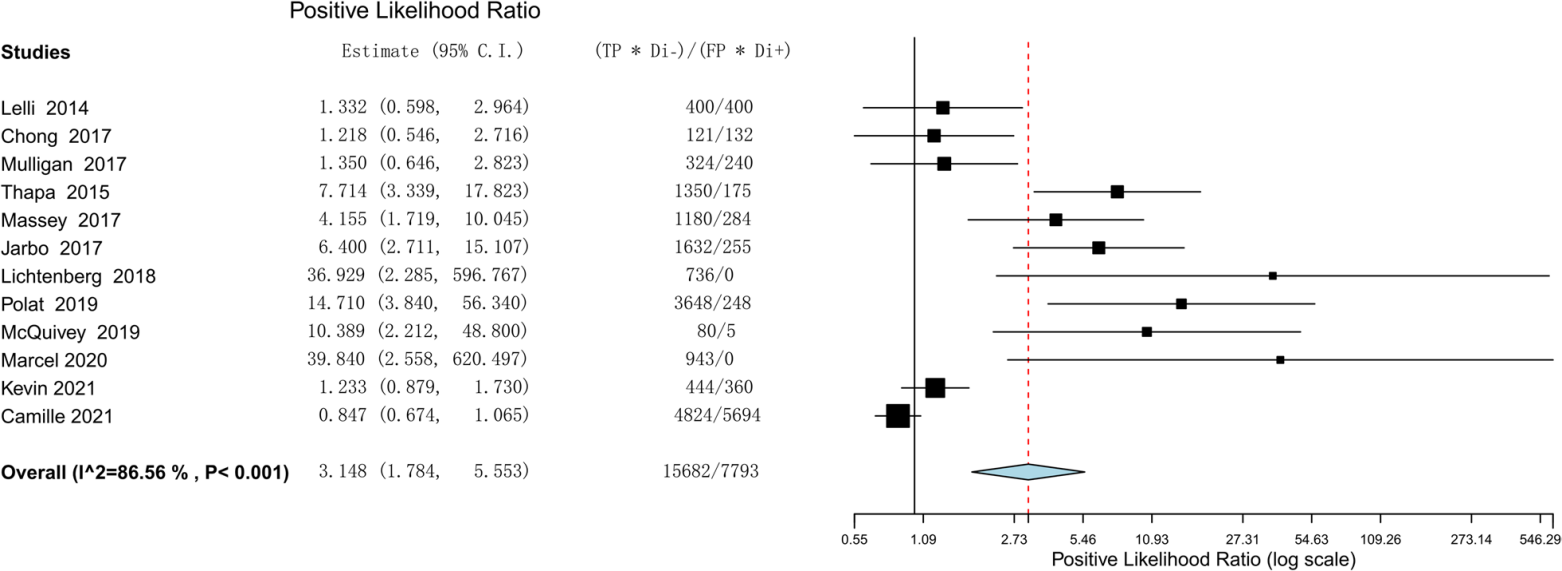
Specificity part of the coupled forrest plot for the lever sign test compared to MRI/ arthroscopy, for the diagnosis of anterior cruciate ligament injuries

**Fig. 5:**
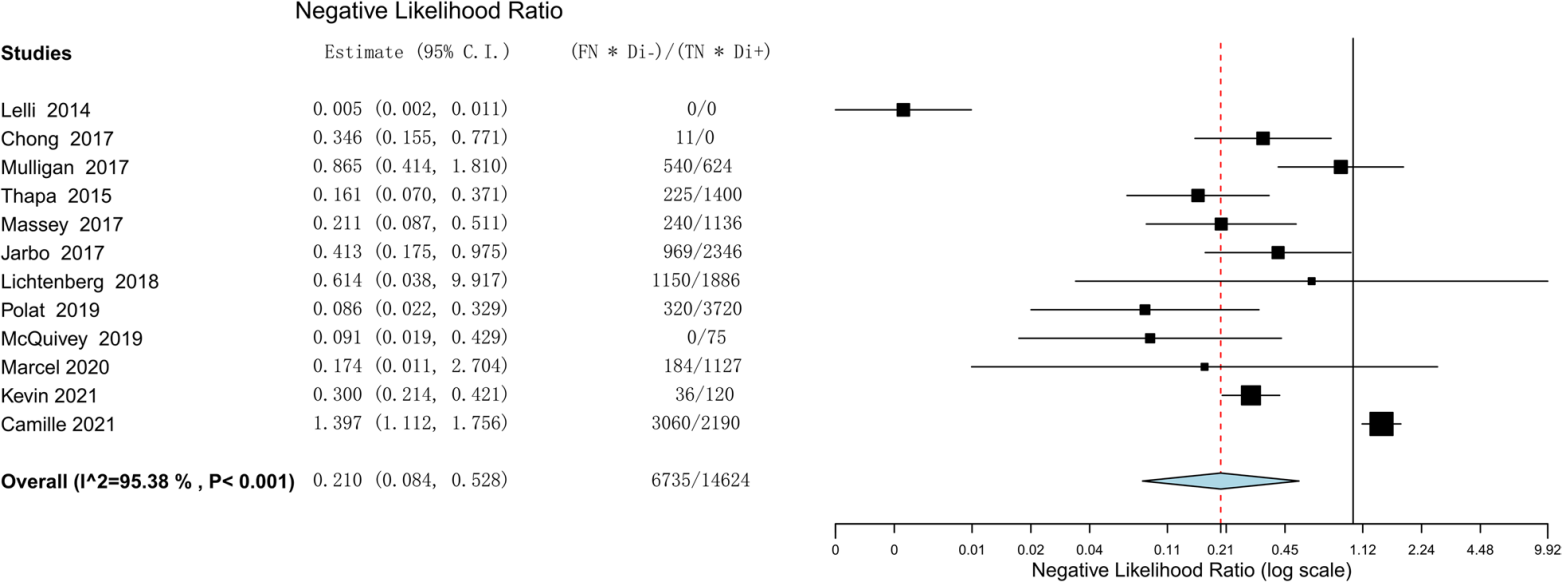
Positive likelihood ratio of the lever sign test to diagnosis anterior cruciate ligament injuries

**Fig. 6:**
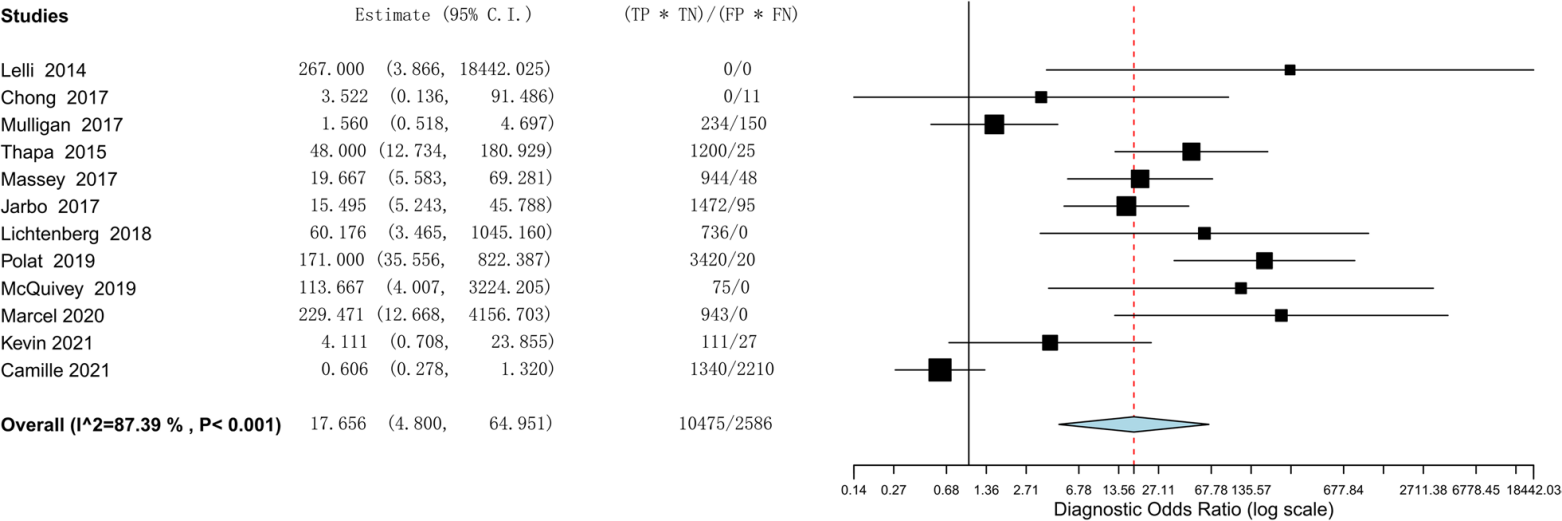
Negative likelihood ratio of the lever sign test to diagnosis anterior cruciate ligament injuries

**Fig. 7:**
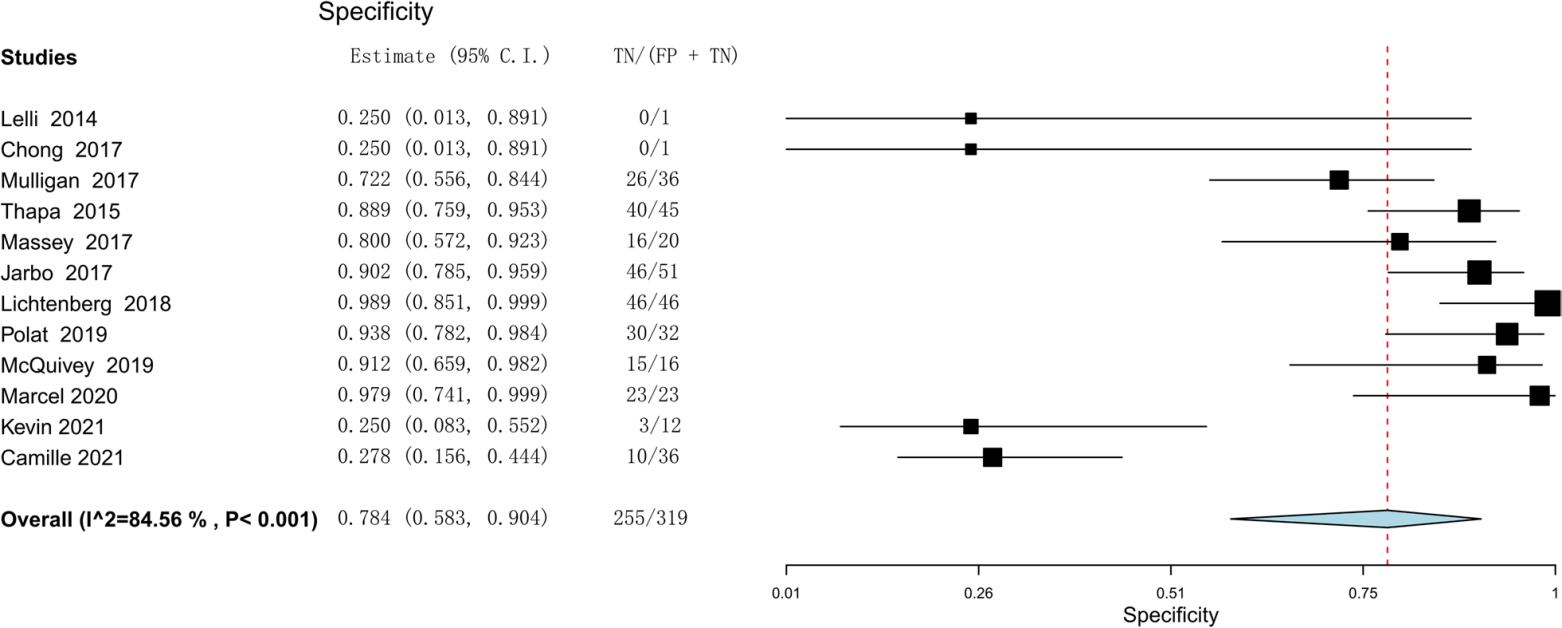
Diagnostic odds ratio of the lever sign test to diagnosis anterior cruciate ligament injuries

**Fig. 8:**
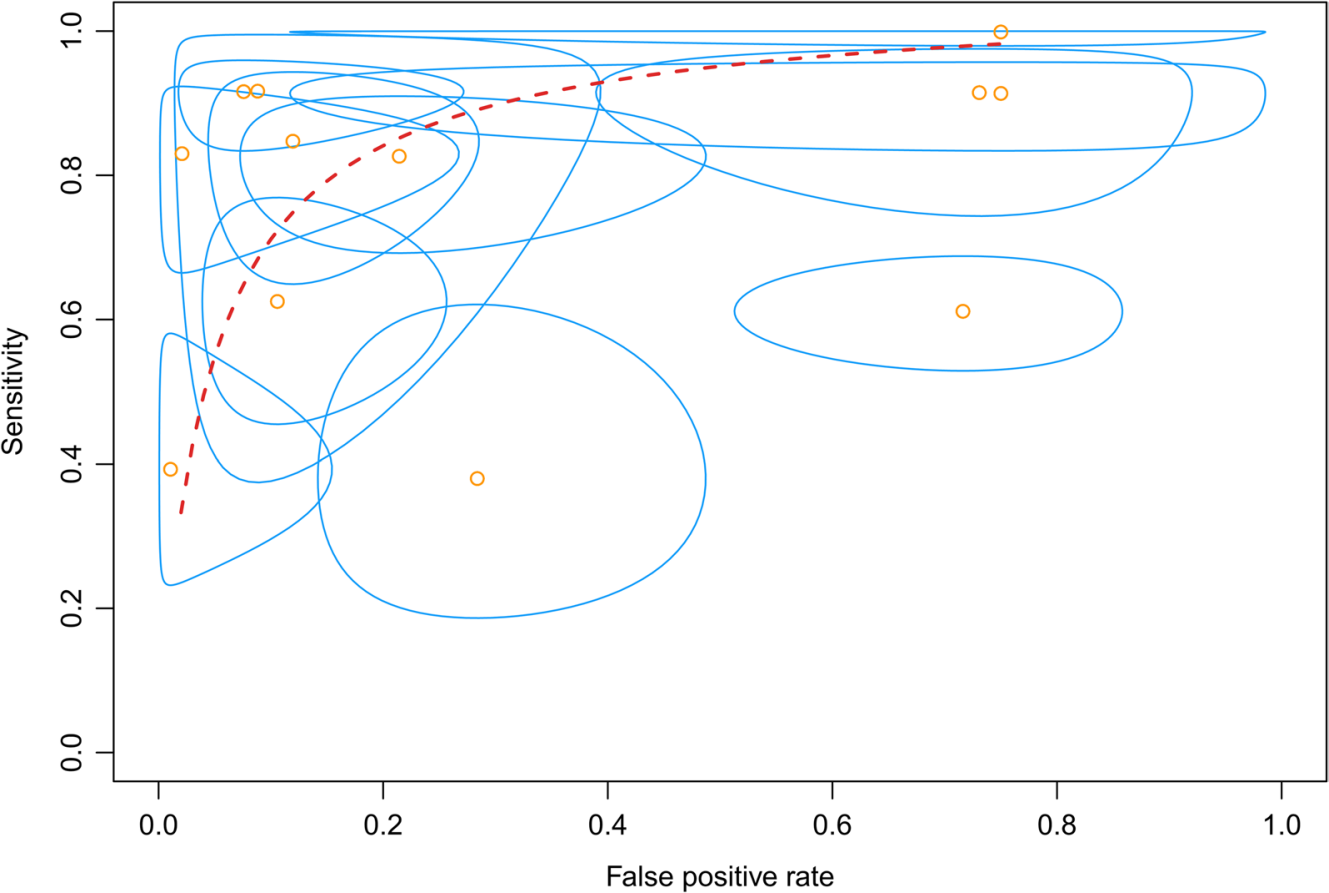
Hierarchical summary receiver operator curve (HSROC) of the lever sign test to diagnosis anterior cruciate ligament injuries

The efficacy of the lever sign examination for diagnosing ACL tears was assessed through the utilization of forest plots illustrating sensitivity, specificity, positive likelihood ratio, negative likelihood ratio, diagnostic odds ratio, and SROC. The pooled sensitivity and specificity of the lever sign test for diagnosing ACL injuries were 0.810 (95% confidence interval [CI] 0.686–0.893) and 0.784 (95% CI 0.583–0.904), respectively. The positive and negative likelihood ratios were 3.148 (95% CI 1.784–5.553) and 0.210 (95% CI 0.084–0.528), respectively. The diagnostic odds ratio was 17.656 (95% CI 4.800–64.951). The AUC of the SROC was 0.912 (95% CI 0.857–0.967). The summary of the meta-analysis results is presented in Figs. 3, 4, 5, 6, 7 and 8.

## Discussion

The ACL is an important stabilizing ligament of the knee joint, and ACL tears are a common injury among athletes and active individuals [24]. Early and accurate diagnosis of ACL tears is crucial for proper management and to prevent further damage to the knee joint [25]. The lever sign test is a simple and widely used clinical test for detecting ACL tears. In this meta-analysis, we evaluated the lever sign test efficacy for diagnosing ACL tears.

The outcomes of the meta-analysis indicate that the lever sign test exhibits a considerable degree of diagnostic accuracy in identifying ACL tears, as evidenced by a pooled sensitivity of 0.810 and specificity of 0.784. The findings are in line with the earlier investigation that has documented the diagnostic accuracy of the lever sign examination in identifying ACL ruptures [26–28]. In a systematic review and meta-analysis of the physical examination tests accuracy for ACL ruptures, Hegedus et al. [29] reported a sensitivity of 0.89 (95% CI 0.82–0.93) and a specificity of 0.96 (95% CI 0.93–0.98) for the lever sign test. The accuracy of physical examination tests for diagnosing ACL tears was assessed in an investigation conducted by Abruscato et al. The lever sign test demonstrated a sensitivity of 0.77 and a specificity of 0.90 [1].

The high sensitivity and specificity of the lever sign test in the meta-analysis suggest that it is a valuable diagnostic tool for identifying ACL tears. However, it is important to note that the diagnostic efficacy of the lever sign test varies widely among studies, which may be due to differences in study design, sample size, and reference standard, the experience and skill of the examiner, the timing of the test (i.e., immediately after injury vs. several days or weeks later), and the presence of other injuries or conditions that may affect the knee joint [30]. It was noted that the diagnostic accuracy of the lever sign examination was higher in studies utilizing arthroscopy as the reference standard compared to those employing MRI. This may be because arthroscopy is considered the gold standard for diagnosing ACL injuries.

It is essential to acknowledge the restrictions of the meta-analysis in order to accurately interpret the outcomes. First, the involved studies exhibited notable heterogeneity, potentially impacting the accuracy estimates. The lack of subgroup analysis concerning study design, sample size, and reference standard etc. is the most prominent deficiency in the present study. Second, the quality of the involved investigations varied, with some studies having a high bias risk. Third, the search strategy may have missed relevant studies, although efforts were made to minimize this risk by using a comprehensive search strategy and by manually searching the reference lists of relevant articles.

## Conclusion

In conclusion, the meta-analysis provides proof of the efficacy of the lever sign test as a valuable diagnostic tool for ACL tears, which is convenient to applied and painless. However, further research is warranted to explore the factors that may influence the lever sign test accuracy, and further systematic review is needed to compare its accuracy with other diagnostic tests for ACL tears, including the Lachman, anterior drawer, and pivot shift tests. Clinicians should also consider the limitations of the lever sign test and use it with other diagnostic modalities to enhance diagnostic accuracy.

## Data Availability

All data included in this study are available upon request by contact with the corresponding author.
