# Correction: Lever sign test for anterior cruciate ligament injuries: a diagnostic meta-analysis

**DOI:** 10.1186/s13018-024-04673-4

**Published:** 2024-04-04

**Authors:** Shiqiang Hu, Xiaoping Wang, Qiyue Wang, Weili Feng

**Affiliations:** 1 Orthopaedics Department, Xiaolan People’s Hospital of Zhongshan, Zhongshan, People’s Republic of China; 2 Sports Medicine Center, Department of Orthopaedics Surgery, First Affiliated Hospital of Shantou University Medical College, Shantou, People’s Republic of China

**Correction: Journal of Orthopaedic Surgery and Research (2024) 19:155**

10.1186/s13018-024-04635-w

Following publication of the original article [1], the author reported that the following authors were omitted from the author group. Shiqiang Hu^1^, Xiaoping Wang^1^, Qiyue Wang^2^ and Weili Feng^1^* have been added to the author group and are presented correctly in this correction article.

The original article [1] has been corrected.

